# A Graph Neural Network Approach for the Analysis of siRNA-Target Biological Networks

**DOI:** 10.3390/ijms232214211

**Published:** 2022-11-17

**Authors:** Massimo La Rosa, Antonino Fiannaca, Laura La Paglia, Alfonso Urso

**Affiliations:** CNR-ICAR, National Research Council of Italy, Via Ugo La Malfa 153, 90146 Palermo, Italy

**Keywords:** graph neural network, deep learning, siRNA, biological networks

## Abstract

Many biological systems are characterised by biological entities, as well as their relationships. These interaction networks can be modelled as graphs, with nodes representing bio-entities, such as molecules, and edges representing relations among them, such as interactions. Due to the current availability of a huge amount of biological data, it is very important to consider in silico analysis methods based on, for example, machine learning, that could take advantage of the inner graph structure of the data in order to improve the quality of the results. In this scenario, graph neural networks (GNNs) are recent computational approaches that directly deal with graph-structured data. In this paper, we present a GNN network for the analysis of siRNA–mRNA interaction networks. siRNAs, in fact, are small RNA molecules that are able to bind to target genes and silence them. These events make siRNAs key molecules as RNA interference agents in many biological interaction networks related to severe diseases such as cancer. In particular, our GNN approach allows for the prediction of the siRNA efficacy, which measures the siRNA’s ability to bind and silence a gene target. Tested on benchmark datasets, our proposed method overcomes other machine learning algorithms, including the state-of-the-art predictor based on the convolutional neural network, reaching a Pearson correlation coefficient of approximately 73.6%. Finally, we proposed a case study where the efficacy of a set of siRNAs is predicted for a gene of interest. To the best of our knowledge, GNNs were used for the first time in this scenario.

## 1. Introduction

Recent advancements in high-throughput technologies have expanded the possibility of analyzing a large amount of biological data. Moreover, biological information increases when considering interactions and relations between different molecules. Many biological systems can be easily represented and modelled as graph networks in which different entities interact with each other through a complex set of interactions or relations. The molecules of a biological network are represented as nodes, and the interactions are represented as edges of the graph [1]. Different types of biological information can be represented in the shape of networks; some examples are protein–protein interaction networks, where interactions are part of a cell signalling cascade inside the cell, different metabolites interacting inside a specific metabolic pathway, and interacting genes belonging to a transcriptional regulative network [2,3]. In this context, artificial intelligence approaches such as machine learning (ML) contribute to revealing hidden relations between molecules inside the cell, thus explaining part of complex biological mechanisms, such as the onset of different diseases. Often, they can efficiently model these interactions for data analysis; for example, for classification or prediction issues, exploiting their relevant characteristics in classification/prediction accuracy. Nevertheless, traditional ML algorithms do not deal with graph-structured data; in this way, they lose the intrinsic relationships regarding the network interaction model. Graph neural networks (GNNs) are recent computational approaches applied to the analysis of data modelled as graph structure [1]. Different from other ML models, which usually work with tabular data, they are able to analyse data represented with a graph structure, such as biological networks. Moreover, they combine the advantage of analysing data, represented with a graph structure, and the feature of modelling the intrinsic topology of data, highlighting the hidden relationships and information between them [3,4]. This provides a simple and intuitive representation of heterogeneous and complex biological processes. Besides this, GNNs are not only suitable for non-Euclidean data but are also able to extract potential graph structures from data without apparent graph structures, such as images, and make inferences based on this structure [2]. A case study of biological data modelled as biological networks is small interfering RNA (siRNA)–messenger RNA (mRNA) interaction networks, where a few mRNAs interact with a large number of siRNAs. Recently, attention has been paid to this biological network due to the ability of siRNA molecules to act in cancer treatment as RNA interference (RNAi) therapeutic agents [5]. In the bio-medical field, siRNA could represent a new potential strategy to modulate cellular mechanisms governing specific pathological states, such as cancer [5,6,7]. They are non-coding RNA molecules acting by binding specific target genes through specific RNA binding site interactions. siRNA efficacy in targeting and silencing a specific gene is linked to the affinity of siRNA to specifically bind an mRNA target, thus defining the gene silencing effectiveness of that siRNA for a specific sequence of the target gene [8]. In this scenario, it is important to predict in silico the siRNA efficacy since the selection of siRNAs with high efficacy values is an important feature for the consequent in vitro siRNA production. Indeed, it allows for the selective binding of the selected target gene with a high affinity, thus blocking its cellular action.

This paper proposes a GNN approach to face the problem of siRNA–mRNA efficacy prediction, defining a specific topological model. Indeed, one of the critical tasks for siRNA–mRNA interaction analysis is the prediction of siRNA efficacy on mRNA binding, which depends upon different siRNA features, such as its sequence feature, thermodynamic feature, secondary structure feature, etc. This can limit the ability of the prediction since these features are biased and incomplete feature vectors. ML has proved to overcome these weaknesses linked to the features of these biological data [9]. The GNN exploits the advantage of directly working with graph-structured data; this way, it does not lose information about the siRNA–mRNA interaction model. The rest of the paper is organised as follows: in Section 1.1, some related works are summarised; Section 3 describes the SiRNA datasets used, the sequence and thermodynamic features, the GNN, and the proposed approach; in Section 2, results, discussion, and a simple case study are reported; finally, Section 4 gives the conclusions.

### 1.1. Related Work

In recent years, many authors used GNNs to solve classification, clustering, and prediction problems in bioinformatics. In [1], the authors discuss, in a comprehensive way, domains in bioinformatics in which GNNs are frequently applied, such as protein function prediction, protein–protein interaction prediction, gene regulatory networks, and in silico drug discovery and development. Authors in [10] propose a GNN-based method for identifying ncRNA–protein interactions (NPIs). The proposed method was compared with four state-of-the-art machine learning methods, obtaining comparable performances but increasing the capability of predicting novel interactions based on network and sequence information. The work in [11] introduces a graph auto-encoder network to create embeddings for scRNA-seq cell data. The results show that modelling the data as a graph increases the flexibility in defining custom features characterizing the genes, the cells, and their interactions. A similar approach for scRNA-seq cell data is described in [12], where, in order to capture the structural information among cells, a GNN module is added to a zero-inflated negative binomial (ZINB) model-based autoencoder. The experimental results on six real scRNA-seq datasets demonstrate that the proposed structural method outperforms state-of-the-art methods in terms of clustering accuracy and scalability. scGNN [13] introduces a graph neural network framework for single-cell RNA-Seq analyses. This framework models heterogeneous gene expression patterns using a left-truncated mixture Gaussian model that aggregates cell–cell relationships with GNNs, and integrates three iterative multi-modal auto-encoders. The results show that the scGNN outperforms existing gene imputation and cell clustering tools on four benchmark scRNA-Seq datasets. In scDeepSort [14], authors designed a pre-trained cell-type annotation method based on a weighted GNN framework. The proposed algorithm consists of three components: (i) the embedding layer, which stores the representation of graph nodes; (ii) the weighted graph aggregator layer, which learns the graph structure information and generates linear separable feature space for cells; (iii) the linear classifier layer, which classifies the final cell state representation. In the weighted graph aggregator layer, a modified version of the GraphSAGE [15] information processing framework was applied as the backbone GNN. From an architectural point of view, in order to consider the edge features and measure the importance of the neighbourhood and the connecting edges, the authors in [16] propose a novel graph neural network named NENN applied for molecular networks. It incorporates node and edge features based on a dual-level attention mechanism, including node-level and edge-level attention.

Regarding the biological problem of siRNA–mRNA efficacy prediction, many machine learning and deep learning algorithms have been applied to solve it; these algorithms are based on the mRNA’s secondary structures and the nucleotides’ specific positions. In [17], a deep neural network that also uses thermodynamic features to characterise the siRNA efficacy of a siRNA–mRNA interaction is proposed. As the best result, the experiments show a Pearson correlation coefficient of 66%, computed between true and predicted siRNA efficacy values. The authors of the papers in [8,18] employ the same kind of thermodynamic features using a linear regression model and obtain similar results. More recently, other studies employ the thermodynamic features and nucleotide positions. In this direction, authors in [9] developed a new siRNA efficacy predictor based on a deep architecture, extracting hidden feature patterns from two modalities, including sequence context features and thermodynamic properties. In [19], authors combine quantitative and qualitative analyses to represent siRNA–mRNA interactions, and, based on this representation, introduce a supported vector regression (SVR) predictor. Finally, authors in [20] propose a hybrid sequence feature-based model, LncMirNet (lncRNA–miRNA interactions network) to predict lncRNA–miRNA interactions via deep convolutional neural networks (CNN). The results show that LncMirNet, compared to six state-of-the-art methods on a real dataset collected from lncRNASNP2 [21], increased the accuracy, area under curve (AUC), and Matthews correlation coefficient (MCC).

## 2. Results and Discussion

### 2.1. Experimental Setup

We performed two kinds of experimental tests. First of all, we carried out a repeated (five times) ten-fold cross-validation procedure, obtaining a total of 50 different predictions for each algorithm. Then, in order to test our model with an independent dataset, we trained the GNN with *dataset_1* and tested it with *dataset_2*. As explained in Section 3.1, in fact, we collected all of the available siRNA datasets, and the only way to test our model with an external test set was considering *dataset_1* and *dataset_2* as the training and test set, respectively. Indeed, both datasets were provided by different research groups, so they can be considered independent of each other. The goodness of the predicted siRNA efficacy was computed using the Pearson correlation coefficient (PCC) [22], as carried out in [9]. PCC is a statistical measure of the linear correlation between two sets of data. In this case, PCC was computed between true and predicted siRNA efficacy values. PCC is in the range [−1; +1], where values close to +1 or −1 indicate a strong correlation (anti-correlation) between the variables, and values close to zero indicate a weak association between the variables. PCC values were then averaged over 50 runs. Moreover, we computed the mean squared error (mse) and the coefficient of determination, also known as *R*-squared or $R^{2}$ [23], which explains what the percentage of the variance of a dependent variable is with respect to an independent variable in a regression model. All of the deep learning models were implemented in Python 3 using the Keras framework [24] with the Tensorflow 2 backend [25]. The proposed GCN model was implemented using StellarGraph library [26], which is a Python library for machine learning on graphs and networks fully integrated with Keras and Tensorflow. Datasets and source code are available at GitHub repository https://github.com/BCB4PM/GNN4siRNA accessed at 1 November 2022.

### 2.2. Model Parameters Fine-Tuning

**Figure 1:**
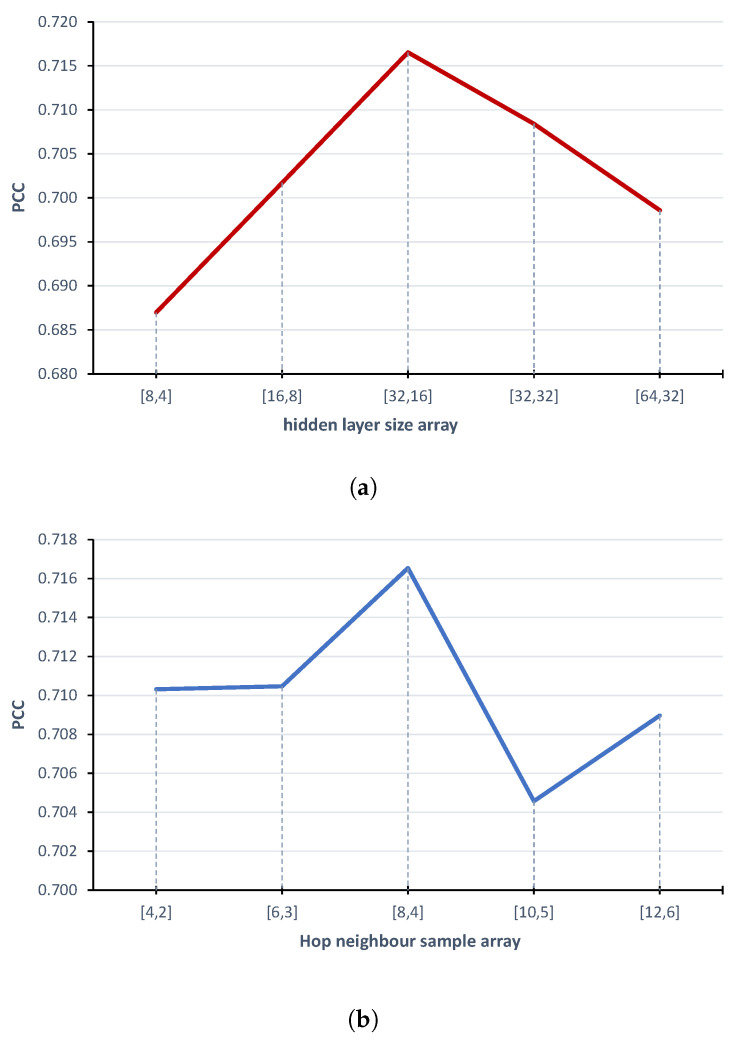
Fine-tuning of (**a**) HinSAGE hidden layers and (**b**) hop neighbour samples hyperparameters with respect to Pearson’s correlation coefficient.

To obtain the best performance from the proposed model, we chose the best number of features for input sequences (siRNA and mRNA) and fine-tuned the model hyperparameters. Table 1 reports the results of our study. With regard to the input sequences, according to the k-mer representation, we tested different *k* values for both siRNA and mRNA genomic sequences to obtain two input matrices with acceptable sparsity. Even though both inputs are genomic sequences, they have mean lengths that differ by at least an order of magnitude; in particular, siRNAs are small sequences, whereas mRNA can have thousands of nucleotides. For this reason, after some tests that we do not report here, we found a similar sparsity when siRNAs and mRNAs are represented with 3-mer (64 features) and 4-mer (256 features), respectively. Regarding model hyperparameters, we performed tests on the batch size, HinSAGE layer sizes, hop neighbour samples, dropout, and optimisation function. Here, we reported details about the most critical parameters of the architecture, i.e., the HinSAGE layer sizes and hop neighbour samples for each layer. Figure 1 shows the fine-tuning of these hyperparameters with respect to the Pearson correlation coefficient. In particular, we first tested different pairs of output neurons for the first and second layer of the HinSAGE architecture (Figure 1a), taking advantage of considering 32 and 16 neurons, respectively. Then, we looked for how many hop neighbour samples for each layer are necessary to reach the best result (Figure 1b), and we obtained eight hops for the first layer and four hops for the second layer.

### 2.3. Experimental Results and Comparison with Other Predictors

**Figure 2:**
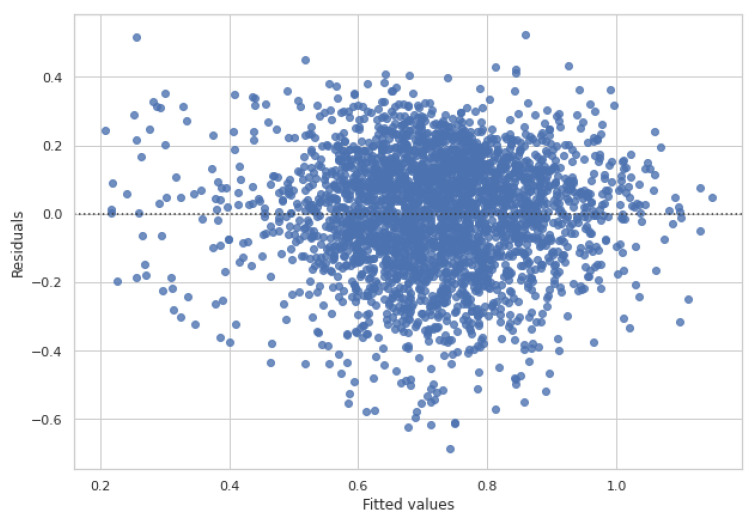
Residuals scatter-plot. Fitted values vs. residuals in *dataset_1*. The dotted line represents the prediction equation.

The experimental results of our GNN approach for siRNA efficacy prediction were computed in terms of the average PCC, mse, and *R*-squared using the parameters summarised in Table 1, as explained in the previous section. Moreover, we estimated regression fits by means of a residual analysis of *dataset_1*. Figure 2 shows how the residuals are randomly scattered around zero; this means that the residuals are consistent with random error, and that the model has no bias in the residuals. Table 2 shows the prediction results of our proposed GNN approach, as well as the results obtained considering other deep learning architecture, as described in Section 3.5. In the upper part of the table, representing the 10-fold cross validation experiments, results are arranged according to *dataset_1* and *dataset_2*, described in Section 3.1. Further experiments on *dataset_3* are summarised and discussed in Supplementary Material and in Figures S1, S2 and Table S1 therein. As expected, all of the algorithms perform better with *dataset_2* with respect to the other dataset. This happens because *dataset_2* has the easiest topology (star graph) and contains only an mRNA target, even though the number of training samples is lower than those of the other datasets. Otherwise, when the complexity of the dataset topology increased, as in *dataset_1*, all of the compared algorithms reached lower results. Interestingly, the proposed method not only reached better results than the other algorithms with all datasets, but was also the most robust concerning the complexity of the topology. Table 2 shows the obtained results in terms of PCC, mse, and $R^{2}$. The proposed GNN outperformed all of the other methods, and the best results of approximately 73% were reached with regard to *dataset_2*. The state-of-the-art method, indicated as the baseline in the table, reached, at most, 69.5% with regard to *dataset_2*. As for the remaining deep architectures, either CNN, LSTM, or DNN did not provide meaningful results. Looking at the performance of the other algorithms, we notice that the baseline algorithm is the second best after the proposed one in terms of PCC and mse. With regard to the $R^{2}$ measure, Table 2 clearly shows how the proposed algorithm reaches higher values with respect to the other approaches. In general, the low $R^{2}$ values, i.e., lower than 0.50, do not surprise us since, in this kind of biological problem, there is an inherent amount of unexplainable variability.

In the lower part of Table 2, we show results obtained during the test phase, i.e., training with *dataset*_1 and testing with *dataset*_2. In this case, although our method reached the best results again in terms of PCC, we noticed a significant drop in performance. This behaviour, however, was not unexpected. As recent literature has demonstrated—see, for example, [27,28], among others—GNNs suffer from poor out-of-distribution (OOD) generalisation capabilities that occur when train and test sets originate from different data distributions. Because *dataset*_1 and *dataset*_2 have very different topologies that reveal different graph measures, such as average clustering coefficient and unique node degrees [27] (Section 3.1), they represent different distributions, and our GNN model is affected by the OOD generalisation issue. In this case, the CNN perform better than the other models in terms of $R^{2}$, obtaining 0.2923 against the 0.2057 reached by our model. Once again, the $R^{2}$ values appear not very significant for this kind of biological problem, since the values are lower than 0.30. In turn, when we considered *dataset*_3, which is the integration of *dataset*_1 and *dataset*_2, both data distributions are learned by the GNN model and, therefore, it is able to generalise when validation data are presented (Table S1).

### 2.4. Biological Application of the Proposed siRNAs Efficacy Prediction Method

siRNAs are part of RNA interference (RNAi) molecules. As previously stated, they can bind target genes in specific sites and block their expression. Translating this concept into clinical practice, they can be used as therapeutic molecules for different pathologies, targeting key genes of specific pathways and re-addressing the altered signalling cascade. They are mostly synthesised by biological methods. Scientific literature shows that many siRNA molecules are already used in clinical trials [29,30,31,32]. siRNA therapeutics has also been tested to prevent or treat various cancers. In this context, the prediction of siRNA efficacy becomes a relevant problem in studies involving RNA interference, as it measures how efficiently the siRNA will silence a specific target gene. It can vary significantly when comparing different siRNA molecules for the same target gene, as a single change in the siRNA sequence can alter its stability for the binding site. Our proposed method, aiming at predicting the efficacy of the interaction between siRNA and mRNA molecules, allows for choosing siRNAs with high efficacy values. Once again, translating this concept into clinical practice, this means that if a siRNA has to be designed and produced to inhibit an essential gene for a specific pathology, the choice of a siRNA with a high efficacy means the use of a molecule that will most likely inhibit the target gene, thus regulating important signalling cascades for that specific pathway. For instance, the Noggin gene (NM_005450) is known to be associated with a poor prognosis of gastric cancer by promoting cell proliferation through EGFR up-regulation [33]. It is also involved in the development of the prostate cancer osteolytic bone metastatic process, which is a common clinical manifestation in advanced-stage patients suffering from prostate cancer [34]. The use of specific siRNA molecules as RNA therapeutics would allow for the inhibition of Noggin’s action, restoring normal EGFR regulation. The silencing of Noggin mRNA by siRNA therapeutics could thus reduce its metastatic potential, promoting partial bone repair in advanced osteolytic lesions and limiting late tumor growth [35]. For these reasons, we decided to use the Noggin gene as a simple case study to test siRNA efficacy prediction for this target. Although Noggin belongs to our knowledge base, it does not influence the rationale behind the proposed case study. Then, we used the OligoWalk Web Server (rna.urmc.rochester.edu/cgi-bin/server_exe/oligowalk), which allows for the design of siRNA molecules for a specific input mRNA sequence. It produced in silico 19 new siRNA molecules, which do not belong to our knowledge base and are candidates for the Noggin gene. We finally predicted the efficacy value of those 19 siRNA molecules. As shown in Table 3, eight siRNAs (siRNA 1, 4, 5, 6, 7, 8, 10, and 16) were predicted to have a high efficacy value of ≥0.80. These molecules could then be selected to be produced in vitro for RNA interference therapy against the Noggin gene.

## 3. Materials and Methods

In this section, we will present the datasets that we used in order to train our model, and then will describe the main characteristics of the deep learning architectures used in this study. A great focus will be given to the graph neural network (GNN) model, and how we chose to represent the siRNA efficacy prediction problem so that GNN could be applied.

### 3.1. SiRNA Datasets

In this work, we tried to collect the siRNA datasets used in the work by [9]. In particular, we kept siRNA interactions, which are siRNA–mRNA pairs and the related efficacy values, provided in the original studies of Huesken [17], Harborth [36], Ui-Tei [37], Vickers [38], and Khovorova [39]. All of those datasets, which we call *dataset_1* in this paper, were originally collected by [40], and we downloaded them thanks to the work by [41]. Finally, we added siRNA interactions provided by [42], called *dataset_2* in this paper. Unfortunately, we were not able to obtain siRNA dataset from siRNADB [43] as achieved in [9] because that database is no longer available. In total, we were able to collect 3518 siRNA sequences and their interactions, referred to as *dataset_3*. The main characteristics of those datasets in terms of number of unique siRNAs, number of unique mRNAs, and number of interactions are summarised in Table 4. We decided to consider *dataset_1* and *dataset_2* separately because the latter has the property to have a single target (mRNA) with many incoming relationships; therefore, from a topological point of view, it represents a star graph. This peculiarity will be investigated in Results section.

### 3.2. Sequence and Thermodynamic Features

In order to train a machine learning model, we need to define and extract some raw features from the input data. In this context, we considered two class of features, namely sequence and thermodynamic features. For the sequence features, we adopted two very popular representation schemes for biological sequences: one-hot encoding and k-mer counting. SiRNA, in fact, are biological sequences that are 21 nt long; each sequence can therefore be represented using a so-called one-hot encoding. Given the four DNA bases A, C, G, T (or U for RNA), each base in a siRNA sequence was mapped to a four-dimensional binary vector: A =[1,0,0,0]; C =[0,1,0,0]; G =[0,0,1,0]; T/U =[0,0,0,1]. Apart from the target site, we also considered its flanking downstream and upstream regions because they affect the efficacy of siRNA [44]. As demonstrated in [9] through experimental trials, we kept 20 nt upstream and downstream the target site, and, therefore, sequences 61 nt long (21 + 2 * 20) were one-hot encoded. If the flanking regions were fewer than 20, the missing bases were encoded with a [0.05,0.05,0.05,0.05] vector, as suggested in [9]. K-mer counting, on the other hand, consists of finding and counting the number of occurrences of a set of fragments of length k inside the original sequence [45]. Given the four-letter alphabet of DNA (RNA), each sequence is therefore represented by means of an integer array of size $4^{k}$. As for thermodynamic features, siRNA efficacy is dependent on two kinds of properties: the thermodynamic stability profile of siRNA duplex [40] and the thermodynamic of siRNA–mRNA interactions [46]. The former was computed following the work of [47]; the latter was obtained through the RNAUp web server tool belonging to the ViennaRNA package [48]. The total number of thermodynamic properties resulted in a 22-dimensional real-valued feature vector.

### 3.3. Graph Neural Networks

Graph neural networks are deep learning architectures that natively deal with graph-structured data. Deep learning and, in general, machine learning algorithms usually work on data belonging to a Euclidean space; however, in recent years, there is an increasing amount of data that can be represented as a network (graph), exploiting, in this way, their intrinsic relationships [3]. In this context, biological data obtained by high-throughput technologies are a relevant example. Many biological processes, in fact, can be represented as biological networks, such as protein–protein interactions and metabolic pathways [1]. Given these premises, it is evident that GNN can be considered as a valid instrument for the analysis of biological networks. A graph G=(V,E) is defined by its set of vertices (nodes) *V*, with |V|=n, and a set of edges (links) *E* (|E|=e), with $e_{ij}\in E$ being an edge between node $v_{i}$ and $v_{j}$. Moreover, a graph can be defined by its adjacency matrix $A\in R^{n\times n}$. In a graph, both nodes and edges can have attributes in the form of a feature matrix $X_{v}\in R^{n\times d}$ and $X_{e}\in R^{e\times c}$, respectively, where *d* and *c* are the dimensionalities of vertex and edge features. If the nodes in the graph represent different entities, e.g., proteins and drugs, we have a heterogeneous graph, and each node type will have its own features. The types of learning tasks on a graph structure can be summarised as follows [1,3]:

- Node classification/regression: in this case, we want to predict a label associated to a node of the graph. That label can be either categorical (classification) or numerical (regression). In a typical scenario, there are labelled and unlabelled nodes in the same graph, so the aim of task classification (regression) is to predict the label of the unknown nodes by means of a semi-supervised learning approach.
- Edge classification/regression: the same scenario as the node classification/regression, but we want to predict a label at edge level.
- Link prediction: in this case, we want to predict if a link exists between two nodes in the graph. In biological networks, in fact, there is often an incomplete knowledge of interactions among biological entities, such as in the protein–protein interaction networks or in the gene regulatory networks (GRNs). As in node classification, a typical scenario adopts a semi-supervised learning approach, where the knowledge of existing edges in the graph is used to predict new connections.
- Graph classification/regression: in this case, we want to predict a label, either categorical or numerical, that characterises the whole graph.

Finally, we need to discuss the type of layers and deep learning algorithms that compose a GNN. Considering the scope of this work, we only focused on graph convolutional networks (GCNs); for a complete taxonomy on GNN, we recommend the survey in [3]. GCNs are a class of GNNs that lend the main concepts of convolutional neural network (CNN) models [49], adapting them to deal with graph data. CNNs are often applied to image processing tasks because they are able to extract spatial features and relationships among pixels, defined into a fixed grid. In the case of graph-structured data, however, CNNs are not directly applicable because the ordering of the adjacency matrix of a graph is not fixed but arbitrary. The most developed GCNs are based on spatial methods, where, during the training process, the embedding of each node in the graph is learnt by merging, through, for example, sum or mean operation, the features of its neighbourhood. Each layer in a GCN, and therefore considers the information coming from a certain neighbourhood up to a fixed radius, with the $l^{th}$ layer that looks at a neighbourhood of *l* radius for each node. The aforementioned ideas have been integrated into the work by [50], where authors introduced the concept of message passing. In their framework, each node and its own neighbour exchangea message with each other, and, thanks to that, each node is able to update its own state, considering the nodes’ features. Then, a readout step at the end of the network merges the node states on the basis of the level of the learning tasks, such as the node level (e.g., node classification), edge level (e.g., link prediction), or graph level (e.g., graph classification).

### 3.4. Proposed Approach

The proposed approach uses a graph-based model to predict the siRNA efficacy in a siRNA–mRNA interaction network. As we previously said, we can easily represent the entities of this problem as the elements of a graph and the prediction problem as one of the learning tasks that the GNN can solve. In more detail, for this biological problem, we have three different elements: (1) siRNA, (2) mRNA, and (3) the siRNA–mRNA interaction. Each of them can be represented by a specific set of features according to its function in the problem. In particular, we considered the aforementioned k-mer counting representation method to create the features of the first two entities, and we took advantage of the thermodynamic features to characterise the siRNA efficacy of a siRNA–mRNA interaction.

**Figure 3:**
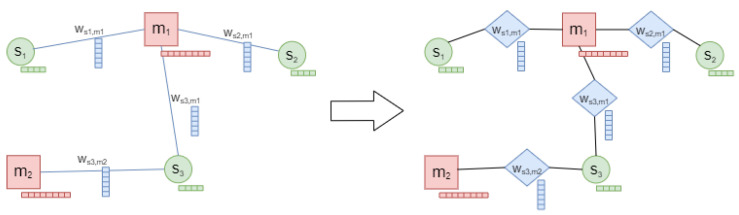
From the standard topology (on the left) to the proposed one (on the right). We replace each interaction edge with three entities: an interaction node (blue diamond) and two edges that connect it to siRNA (green circle) and mRNA (red square), respectively.

Intuitively, the easy way to represent these entities within a GNN model is to consider an undirected heterogeneous graph with two kinds of nodes (i.e., siRNA and mRNA) and one kind of edge (i.e., siRNA–mRNA interaction). In this case, we could solve the siRNA efficacy regression problem with the edge regression task that we introduced before. As shown on the left of Figure 3, this graph will contain a siRNA node $s_{i}$, with i=1,2…,z, where *z* is the number of siRNAs, which is defined with a feature vector containing the k-mer representation of the *i*-th siRNA sequence; in addition, there will be an mRNA node $m_{j}$, with j=1,2…,p, where *p* is the number of mRNAs, which is defined with its proper feature vector containing the k-mer representation of the *j*-th mRNA sequence. At this point, a unique type of edge, the $w_{s_{i},m_{j}}$, represents the interaction between the *i*-th siRNA and the *j*-th mRNA. Each edge has a weight value (the siRNA efficacy) and a vector containing thermodynamic features. Unfortunately, the GCN models cannot exploit all of the entities that we arranged in this topology since the message-passing mechanism only catches information by considering graph node features and graph topological structural information. This means that this kind of model does not fully incorporate edge features into this mechanism, but uses them as filters to multiply the node feature matrix [51]. In biological networks, the interactions often contain information closely related to the entity to which they are connected, sometimes representing a part of the entity itself. In this case study, for example, the thermodynamic features are strictly related to the siRNA entity and define a piece of crucial knowledge that must be modelled if we want to study this biological problem. In addition, to demonstrate that using edge features as filters is insufficient for solving this problem, we performed a preliminary study with this topology representation that we do not report in this work; according to our premises, the obtained results were of poor quality.

For all of those reasons, we chose to transform the proposed graph topology into a new topology, where we translated the information of an interaction edge within a new interaction node of the graph. As reported on the right of Figure 3, we introduced three new entities that replace the interaction edge: a new kind of node, which we call the interaction node (the blue diamond in the figure), and two edges that connect the interaction node with siRNA and mRNA, respectively. The features of the interaction edge become the features of the interaction node, and the weight of the original interaction edge is now the label of the interaction node. The new edges that we introduced have no properties and only perform a structural function. Using the proposed graph topology, we can face the siRNA efficacy regression problem with a node regression task. Conversely to what other literature approaches, such as [16], suggested, i.e., a framework composed of dual-level attention layers used to consider both nodes and edges features, we preferred the proposed enriched topology that introduces, for this kind of problem, a lower complexity with respect to the first representation that we introduced (the left of the Figure 3). In fact, from the point of view of the graph adjacency matrix, it only introduces |E| additional elements, with $\frac{e}{2}\leq \left|E\right|\leq e-1$, where |E| is equal to the number of interaction edges. To efficiently perform the node prediction task on the proposed topology, we chose to exploit one of the most flexible implementations of the GCN algorithm, i.e., the GraphSAGE platform [15]. This implementation uses an inductive approach that leverages spatial-based attribute information to train embeddings for unseen test nodes with a high accuracy. It exploits a latent vector representation to perform an aggregation strategy, where features of each node are fused with features of neighbour nodes. This strategy uses a two-layer architecture, where a node from the graph calculates its representation by exploiting the aggregation of its neighbours’ representations calculated in the previous layer. In particular, we used an extension of the GraphSAGE approach, the HinSAGE (Heterogeneous GraphSAGE) algorithm [26], which can take into account the heterogeneity of nodes and edges in the graph.

**Figure 4:**
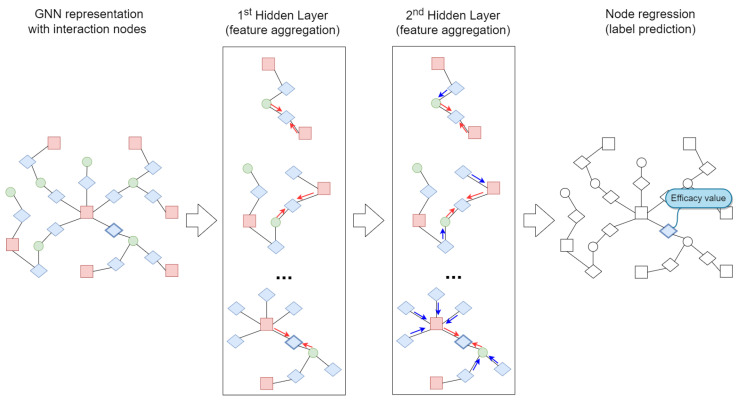
Architecture for siRNA efficacy value prediction. Each HinSAGE hidden layer performs a node feature aggregation among neighbours. For each query node, such as the bold shape interaction node, red arrows aggregate neighbours’ features, and blue arrows aggregate the neighbour neighbours’ features.

Figure 4 reports a schema of the HinSAGE architecture for the proposed approach. This architecture exploits two hidden layers to predict the label of the afore-defined interaction nodes, i.e., the efficacy value of the siRNA–mRNA interactions. For each layer, it is necessary to specify the number of hops that define the node neighbourhood radius. In the first hidden layer of the figure, we considered a hop to equal 1. Red arrows show the direction of the feature aggregation. We used the same neighbourhood depth for the second hidden layer; here, the nodes’ feature aggregation considers the neighbours’ representations calculated in the previous layer (blue arrows). In Figure 4, as an example, we predict the siRNA efficacy value of the bold shape interaction node on the left of the figure using the HinSAGE two-layer architecture and a node regression strategy.

### 3.5. Baseline Models

We considered several deep learning models as a baseline for comparison with our approach. First of all, we chose the state-of-the-art contribution; that is, the work by Han and colleagues [9]. In that work, they implemented a 2D CNN that takes, as an input, the one-hot representation of the siRNA target sites, including their flanking regions (see Section 3.2). Then, they concatenated the thermodynamic features with the feature extracted by the CNN after a pooling operation. Finally, a feed-forward neural network was stacked, with a single unit output layer (sigmoid activation) that provides the siRNA efficacy value. The original characteristic of the model in [9] is that they implemented 15 convolution kernels of different sizes, and then merged the features obtained by the convolution operations. Since the source code is not available, we re-implemented their deep model. The other deep learning models used for comparison were based on different architectures, including a 1D CNN, long short-term memory (LSTM) network, and dense neural network (DNN). The CNN model was composed of three 1D CNN layers, taking, as an input, the one-hot representation of the siRNA target sites, including their flanking regions, each one followed by a max pooling layer. Then, after a flattened layer, the thermodynamic features were merged and the resulting features were fed to a feed-forward network, composed of two dense layers with ReLU activation. The output was a single node with linear activation. In order to avoid overfitting, dropout layers were inserted after the first two max-pooling layers, as well as after the two dense layers of the feed-forward part of the network. LSTM [52] is a type of recurrent neural network (RNN) that takes into account both short-term and long-term memory components by means of a feedback mechanism that loops back previous outputs as a sort of memory. LSTM networks are particularly suited to work with sequence data and time series. For that reason, we applied an LSTM network to analyse siRNA sequences. Our LSTM model took, as an input, the one-hot representation of the siRNA target sites, including their flanking regions, and it was composed of two LSTM layers; after that, the thermodynamic features were included and the resulting features were fed into a feed-forward network composed of two fully connected layers, with ReLU activation. The output was a single node with linear activation. Dropout layers were inserted after the first LSTM layer and after the two dense layers in the feed-forward part of the network. The DNN model that we adopted was made of three stacked fully connected (dense) layers, each with ReLU activation and followed by a dropout layer. This architecture took, as an input, the concatenation of the k-mer representation of the siRNA target sites, including their flanking regions, with the corresponding thermodynamic features. The output was once again a single node with linear activation.

## 4. Conclusions

Many biological mechanisms can be explained as interaction networks among biological entities. In this context, GNNs represent a class of very suitable tools for dealing with graph-structured data. In this paper, we proposed a GNN approach, based on a supervised implementation of a GCN, that is able to analyse the interaction between siRNA and mRNA molecules, with the aim to provide a prediction of the efficacy value of these interactions. GNNs were used for the first time in the proposed scenario. We defined a graph model that takes into account features belonging to siRNA, mRNA, and their interaction, and we set up a node regression task using a modified version of a GraphSAGE layer that deals with heterogeneous graphs, namely HinSAGE. Our GCN approach was compared, in terms of the PCC of the predicted efficacy values and mse, with the state-of-the-art model, and also with other well-known deep learning architectures, including CNN, LSTM, and DNN. Using a five-time repeated 10-fold cross-validation procedure against three datasets with different graph topologies and graph sizes, we obtained that the proposed method outperformed all of the other computational models in terms of PCC and mse. Moreover, it appears more robust than the other ones at varying datasets since it provides the lowest standard deviation among the PCCs of the three datasets. In conclusion, our GCN approach demonstrated its effectiveness when dealing with biological graph-structured data, with respect to other deep learning models that, on the other hand, did not consider the inner structure of the input data.

## Figures and Tables

**Table 1:** Parameters and hyperparameters of the input sequences and GNN model, respectively. The same values were used for all datasets, except for the Adamax learning rate, where * refers to *dataset_2* and ** refers to the other two datasets.

	Parameters	Values
**Input** **sequences**	*siRNA k-mer size*	3
	*mRNA k-mer size*	4
	*number of thermodynamic features*	22
**GNN** **model**	*batch size*	60
	*hop neighbour samples*	[8, 4]
	*HinSAGE layer sizes*	[32, 16]
	*dropout*	0.15
	*Adamax learning rate*	0.005 */0.001 **
	*loss function*	mean squared error

**Table 2:** Comparison of the proposed GNN approach against the other four models in terms of PCC, mse, and $R^{2}$. Both 10-fold cross validation and external testing are reported. Bold values indicate best results.

			Algorithms				
			Proposed GNN	Baseline	CNN	LSTM	DNN
**10-fold** **Cross Validation**	**dataset_1**	PCC	**0.6918**	0.5554	0.4996	0.4428	0.4723
		mse	**0.0253**	0.0336	0.0455	0.043	0.0403
		$R^{2}$	**0.4459**	0.2817	0.0243	0.0854	0.0275
	**dataset_2**	PCC	**0.7361**	0.6959	0.4975	0.4864	0.4797
		mse	**0.0204**	0.0228	0.0422	0.0351	0.0441
		$R^{2}$	**0.4882**	0.4674	−0.0124	0.1484	−0.0302
**External Testset**	**dataset_1 vs. dataset_2**	PCC	**0.5740**	0.5258	0.5477	0.5323	0.4744
		mse	0.0336	0.0351	**0.0299**	0.0325	0.0337
		$R^{2}$	0.2057	0.1741	**0.2923**	0.2313	0.2042

**Table 3:** Predicted efficacy values for a set of Noggin siRNA candidates designed with the OligoWalk algorithm.

siRNA Candidates for the Noggin Gene		Predicted Efficacy Value
siRNA	Sequence	
1	UUUGAUCUCGCUCGGCAUG	0.8078
2	AUGUGGAGAUAGUGCUGGC	0.7686
3	AAUGUCUGCGACCACAGCC	0.7858
4	UUCAGAUCCUUUUCCUUGG	0.8317
5	AACUCUAGCCCUUUGAUCU	0.8453
6	AGCCACAUCUGUAACUUCC	0.8106
7	AACUUCCUCCGCAGCUUCU	0.8324
8	AUCUGUAACUUCCUCCGCA	0.8408
9	UUUUCCUUGGGGUCAAAGA	0.7826
10	AUGAAGCCUGGGUCGUAGU	0.8261
11	UCAGAUCCUUUUCCUUGGG	0.7641
12	UUCUUGCCCUGGGCCAAGC	0.7843
13	ACGUAGCGCGGCCAAAAGC	0.7482
14	AGAAUGUCUGCGACCACAG	0.7767
15	ACCUUCACGUAGCGCGGCC	0.7248
16	AAAGAUAGGGUCUGGGUGU	0.8055
17	AAAAGCGGCUGCCCAGGUC	0.6789
18	AGAUAGUGCUGGCCGCCGG	0.7341
19	AGAUCCUUUUCCUUGGGGU	0.7616

**Table 4:** Number of siRNAs, mRNAs, and interactions for each dataset.

Dataset	N. of siRNAs	N. of mRNAs	N. of Interactions	Ref.
*dataset_1*	2816	44	2816	[40,41]
*dataset_2*	702	1	702	[42]
*dataset_3*	3518	45	3518	[9]

## Data Availability

The data and algorithms presented in this study are openly available in GitHub repository at https://github.com/BCB4PM/GNN4siRNA, accessed at 1 November 2022.

## Supplementary Materials

The following supporting information can be downloaded at: https://www.mdpi.com/article/10.3390/ijms232214211/s1.

## Abbreviations

The following abbreviations are used in this manuscript:

GNN	Graph neural network
ML	Machine learning
siRNA	Small interfering RNA
NPI	ncRNA–protein interaction
ZINB	Zero-inflated negative binomial
AUC	Area under curve
MCC	Matthews correlation coefficient
GRN	Gene regulatory network
GCN	Graph convolutional network
CNN	Convolutional neural network
LSTM	Long short-term memory
DNN	Dense neural network
RNN	Recurrent neural network
PCC	Pearson correlation coefficient
OOD	Out-of-distribution
