# Supplementary material for: A Graph Neural Network Approach for the Analysis of siRNA-Target Biological Networks
Source: Int J Mol Sci. 2022 Nov 17;23(22):14211. doi: 10.3390/ijms232214211 (PMC9696923; doi:10.3390/ijms232214211)
Supplement: Supplementary file 1 [file ijms-23-14211-s001.zip › ijms-1942412-supplementary.pdf]

Supplementary Material

Experimental test on dataset_3

In this supplementary material, we are going to show the results obtained from a repeated (5 times) ten-fold cross validation procedure over *dataset_3*, which is the merge of *dataset_1* and *dataset_2* First of all, we looked at the goodness of the training phase, therefore for a complete ten-fold cross validation procedure of the full dataset, we plotted the loss (mse) vs the number of epochs, for both training and validation sets, as shown in Figure S1. There, we can notice that in about 8 out of 10 folds, train and validation losses have the same trend, with the two curves almost overlapped. That means the training was successful and without overfitting issues. Only in two cases, Figure S1(d) and Figure S1(d)(e), the train loss and the validation loss diverge, that means that the network overfitted, and it was not able to generalise when dealing with the validation set. Figure S2 shows how the residuals are randomly scattered around zero; that means the residuals are consistent with random error, and the model has no bias in the residuals. In Table S1, we show the prediction results in terms of PCC, R-squared and mse. Once again, our GNN approach outperformed all the other methods, reaching a PCC score of about 71.6%.

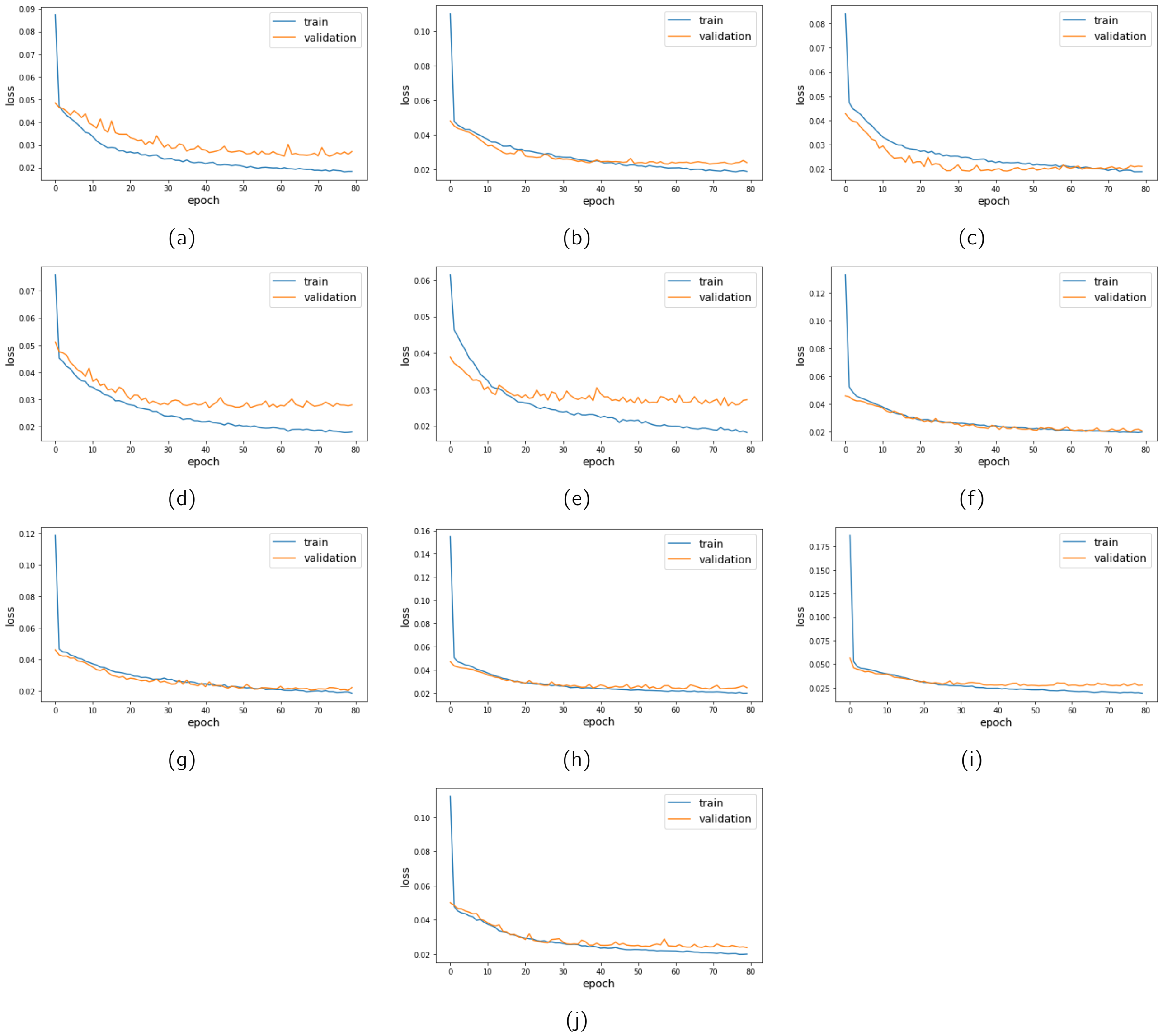

Figure S1: Loss values vs number of epochs during the model training progress. One plot for each fold of the ten-fold cross-validation with *dataset_3*.

Table S1: 10-fold Cross Validation on *dataset_3*. Comparison of the proposed GNN approach against the other four models in terms of PCC, mse and R^2. Bold values indicate best results.

			**Algorithms**				
			**Proposed GNN**	**Baseline**	**CNN**	**LSTM**	**DNN**
**10-fold Cross Validation**	**dataset_3**	PCC	**0.7165**	0.6208	0.561	0.5306	0.5356
		mse	**0.0252**	0.0312	0.0454	0.0393	0.0408
		R^2	**0.487**	0.3685	0.0675	0.1878	0.1737

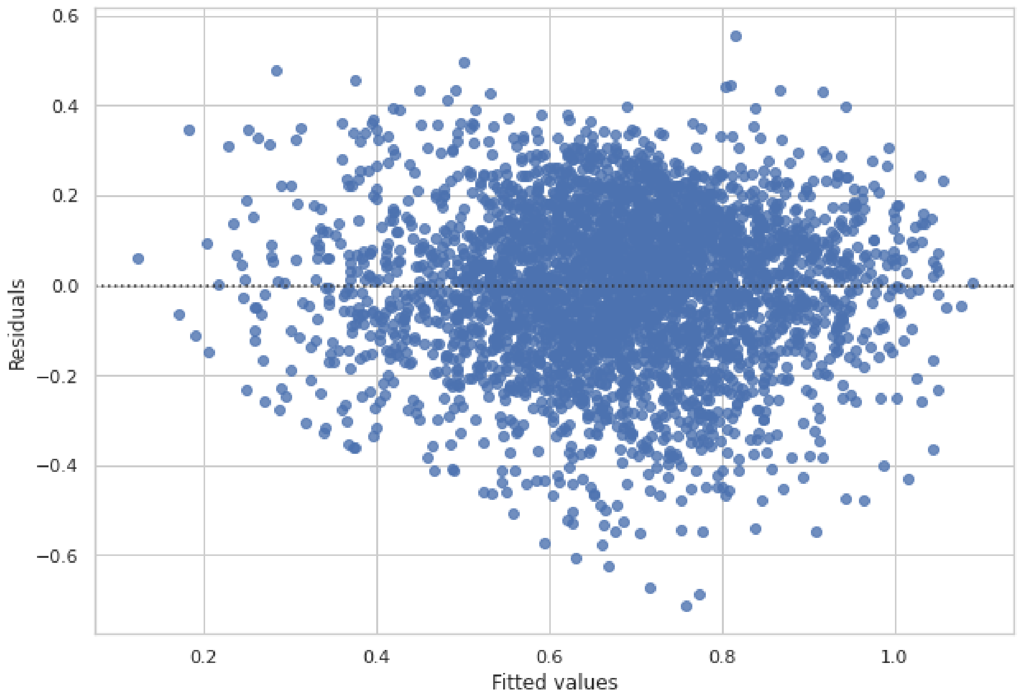

Figure S2: Residuals scatter-plot. Fitted values vs. residuals in *dataset_3*. The dotted line represents the prediction equation.
